# A Case Report of a Pregnancy Complicated by Sigmoid Volvulus in the Extreme Preterm Gestational Age

**DOI:** 10.1002/ccr3.70110

**Published:** 2025-01-16

**Authors:** Naghmeh Kian, Atefeh Moridi

**Affiliations:** ^1^ School of Medicine Shahid Beheshti University of Medical Sciences Tehran Iran; ^2^ Preventive Gynecology Research Center Shahid Beheshti University of Medical Sciences Tehran Iran

**Keywords:** intestinal obstruction, OB & GYN, pregnancy, volvulus

## Abstract

Intestinal obstruction is a rare but life‐threatening incidence in pregnancy. Diagnosis can be challenging for clinicians as the symptoms might be approached as other common obstetric complications. Performing radiological and abdominal surgery are also areas of great concern in this field; since radiologic studies inevitably expose the fetus to radiation and the treatment options mostly involve surgery that is worrisome during gestation. The maternal and fetal outcomes are dependent on timely diagnosis and management; as intestinal perforation, necrosis and peritonitis can happen and lead to fetal or maternal mortality or morbidity. In this study, we present a challenging case of a 36‐year‐old pregnant woman with severe abdominal pain and distension that emergently underwent surgery after the diagnosis of sigmoid volvulus without the gestation being discontinued. To our knowledge, our study presents one of the rarest cases of intestinal obstruction during pregnancy that was managed surgically without pregnancy termination. Further we will discuss intestinal obstruction in pregnancy based the current literature.

**Summary**

- Intestinal obstruction including sigmoid volvulus is an unusual incident during pregnancy, which might be mismanaged due to the lack of clinical suspicion or delayed action.
- This study provides useful information in regards to timely diagnosis and practical methods of management in order to prevent gestational morbidity and mortality.

## 1 Introduction

Obstruction of the intestinal lumen is uncommon during pregnancy and is reported to happen from one in 1500 to one in 66,431 pregnancies [1]. Since first described by Dr. Braun in 1885, Sigmoid volvulus is known as a common type of intestinal obstruction and is reported in 44% of pregnant cases with intestinal obstruction [2, 3]. In the first, second, and third trimesters, sigmoid volvulus reportedly happened in 6%, 19%, and 54% of cases, and in 21% in the postpartum period [4].

The most common causes of intestinal obstruction are adhesion, volvulus, intussusception, carcinoma, hernia and appendicitis [4]. The clinical presentation during pregnancy might be obscure due to the physiological changes of maternal body, thus making quick diagnosis and timely management a challenge [5]. With that being said, intestinal obstruction is a diagnosis that can be made when high amount of clinical suspicion is present or otherwise it will be missed and might lead to bowel ischemia, necrosis, perforation, peritonitis, sepsis, preterm labor, and both fetal and maternal death [6].

The decision to perform a radiological study (e.g., a CT scan in our case) is often not favorable during pregnancy due to the concerns for fetal radiation exposure. However, an abdominal contrast‐enhanced CT scan, which is available in most centers including ours, provides a definitive diagnosis in a short amount of time and determines the exact site of obstruction for further intervention [7, 8]. The type of intervention and the decision to finish the pregnancy is dependent on the gestational age, clinical scenario, the extent of bowl ischemia and the viability of the fetus.

In this study, we present a 36‐year‐old multiparous woman in her 24th weeks' gestation who was diagnosed with sigmoid volvulus after an oral contrast‐enhanced abdominal CT scan and underwent a Hartman procedure emergently without the pregnancy being discontinued.

## 2 Case History/Examination

Our case was a 36‐year‐old multiparous woman in her 24th weeks' gestation who came to the emergency room of Mahdieh OB & GYN hospital, complaining of abdominal pain. The patient had an uneventful pregnancy until 3 days ago when she started feeling an abdominal pain and pressure, which increased in severity day by day. She was unable to defecate and pass gas in the last 3 days. She has had visited several doctors for her constipation and received plenty of bisacodyl prescriptions which did not change her condition.

She had given birth to four healthy kids and had no history of abortion or fetal death. The first two deliveries were performed vaginally but the last two children were delivered by cesarean section due to breech presentation and repeated cesarean, respectfully. Her last pregnancy dated back to 6 years ago. The patient did not receive any prenatal care during this pregnancy.

Except for bisacodyl suppositories in the last 3 days, the patient was taking no medications other than routine supplements. She declared no past medical condition and no history of surgery other than the cesarean sections.

## 3 Management

The patient was an ill‐appearing woman who walked into the OB & GYN emergency room with her abdomen protruding. Her abdomen was obviously distended and in severe pain as she described. She had no complain of vaginal bleeding or discharge. Her vital signs were stable and her BMI was in normal range.

On physical examination, the abdomen was significantly distended and slightly tender on palpation and the examination for rebound tenderness and guarding were negative. The scar of a fan incision was evident in her lower abdomen. The bowel sounds were not heard on auscultation. Vaginal exam with speculum showed a multiparous cervical orifice with no abnormal finding which ruled out premature rupture of membranes. The possibility of uterine rupture was also assessed and ruled out through abdominal examination and further ultrasonography.

The laboratory findings showed leukocytosis (WBC: 14,200 with 90% neutrophil), normal hemoglobin and platelet as well as normal renal and liver function tests and urinalysis. In the emergency room, the fetal heart pulsation was detected via ultrasonography and the amniotic fluid showed to be adequate; the fetal biometry was consistent with gestational age and there was no evidence of placental abruption.

**FIGURE 1:**
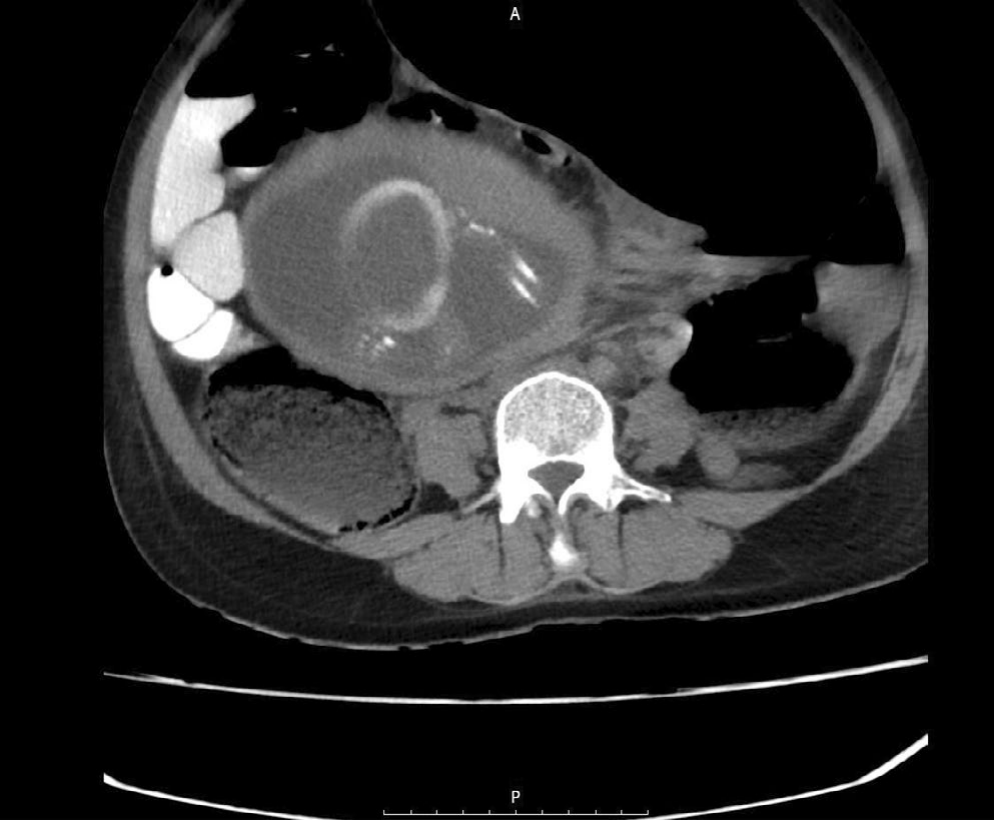
Radiological studies Figure 1 (transverse view) show largely distended bowel loops as well as contrast compaction in one loop that clarifies the presence of a bowel obstruction next to the gravid uterus.

**FIGURE 2:**
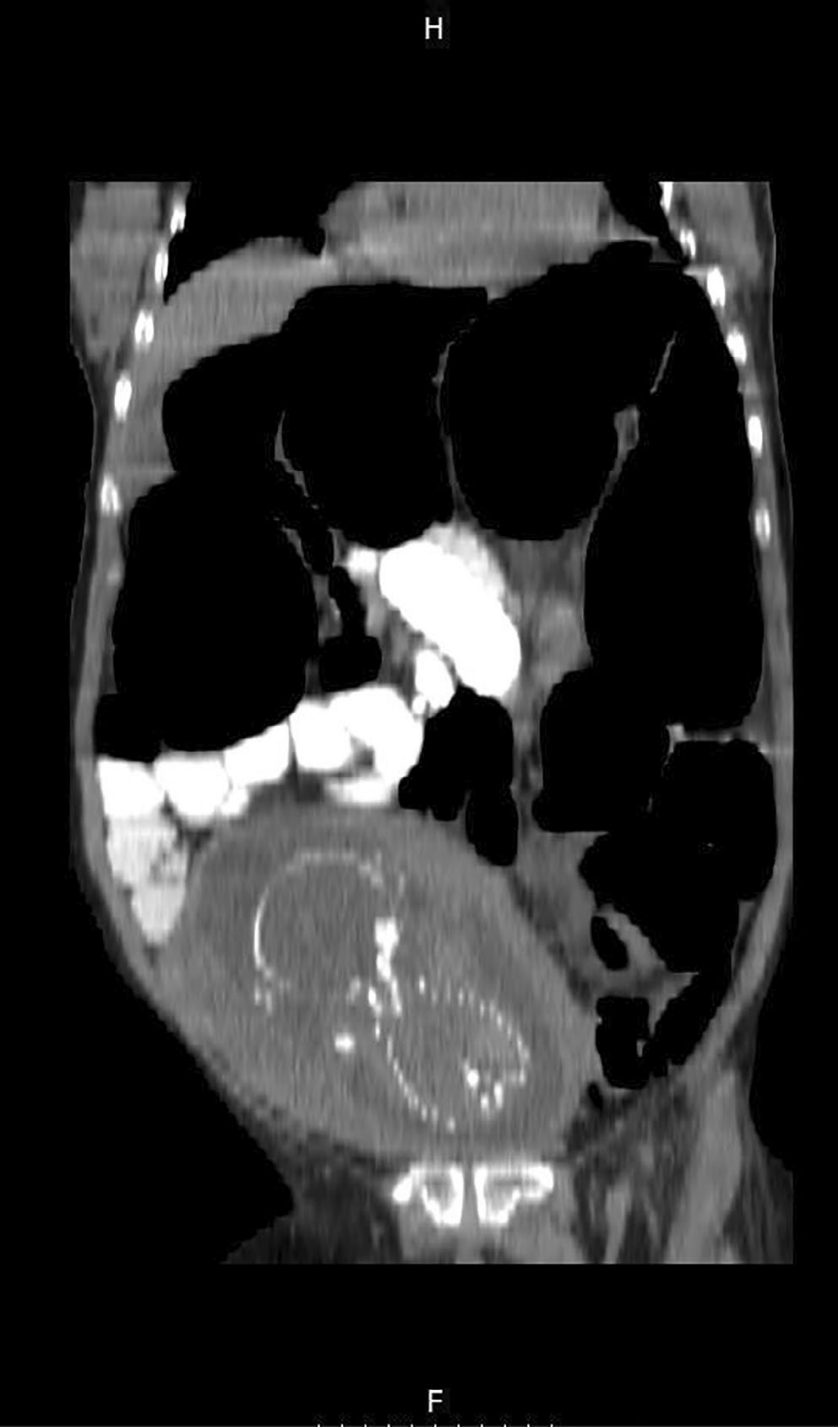
(Coronal view) shows the gravid uterus in proximity to dilated loops of colon and a transition point in which contrast is compacted.

**FIGURE 3:**
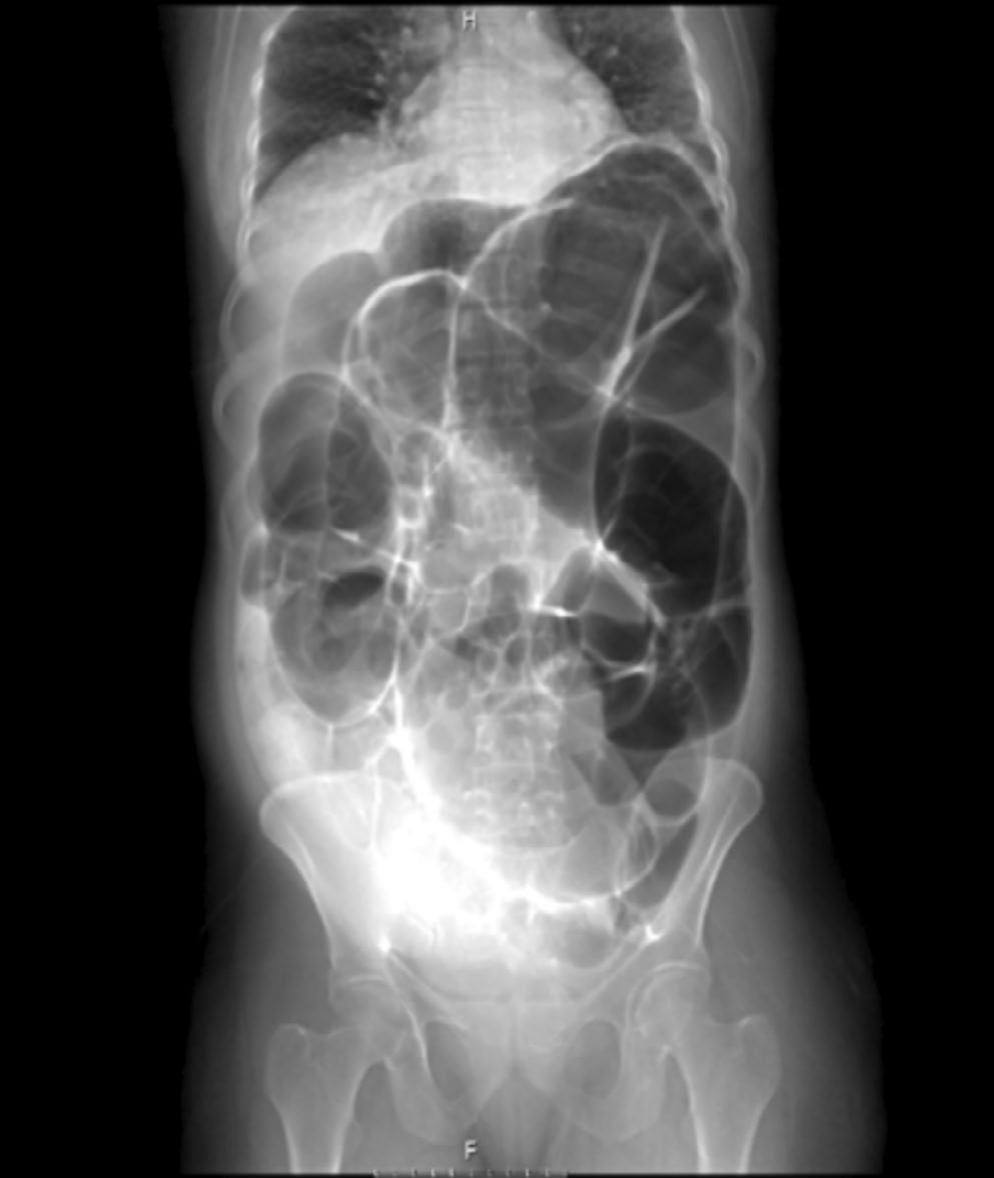
Shows an abdominal radiograph with the classic coffee‐bean sign.

**FIGURE 4:**
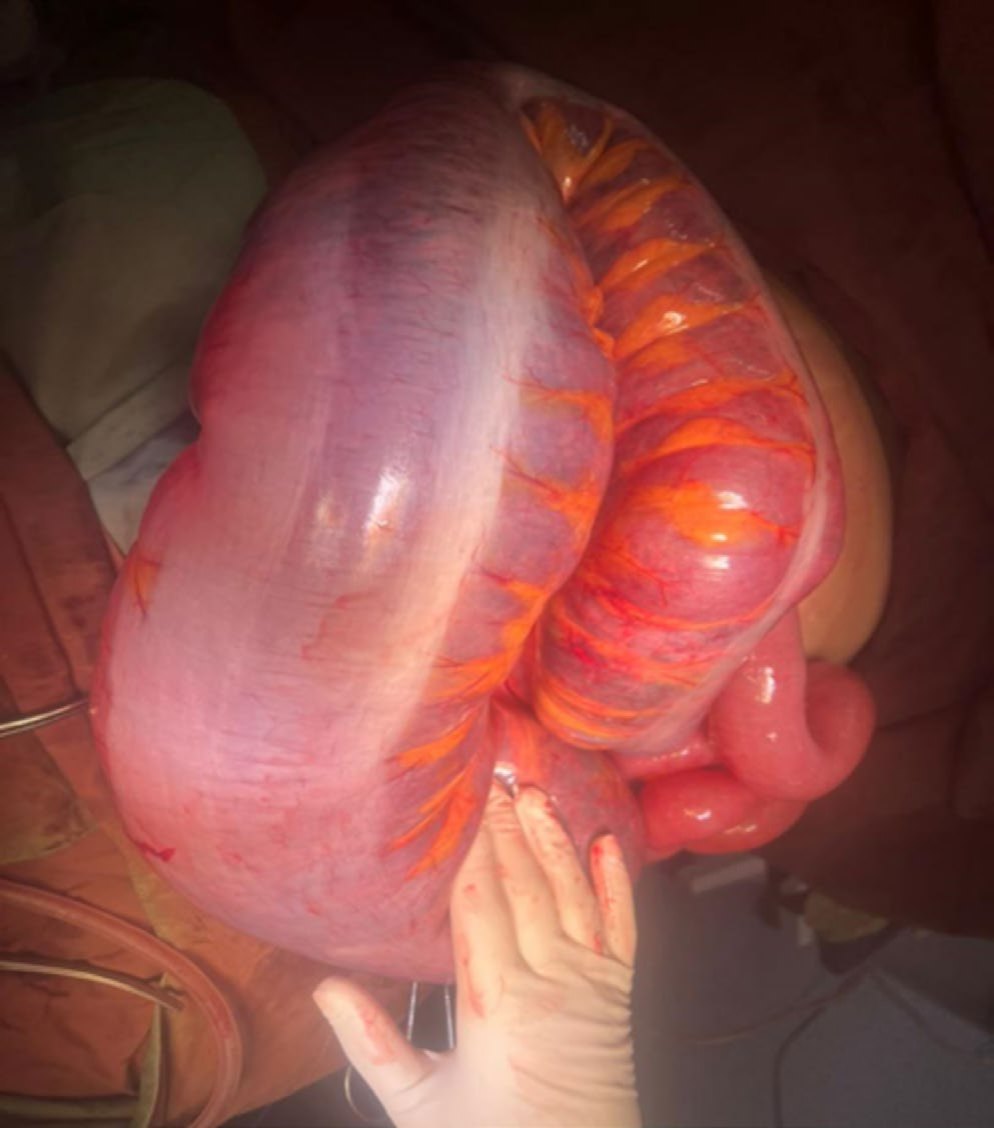
Demonstrates the dilated loop of sigmoid colon, which were found next to the gravid uterus and match the clinical and radiologic findings of sigmoid volvulus.

**FIGURE 5:**
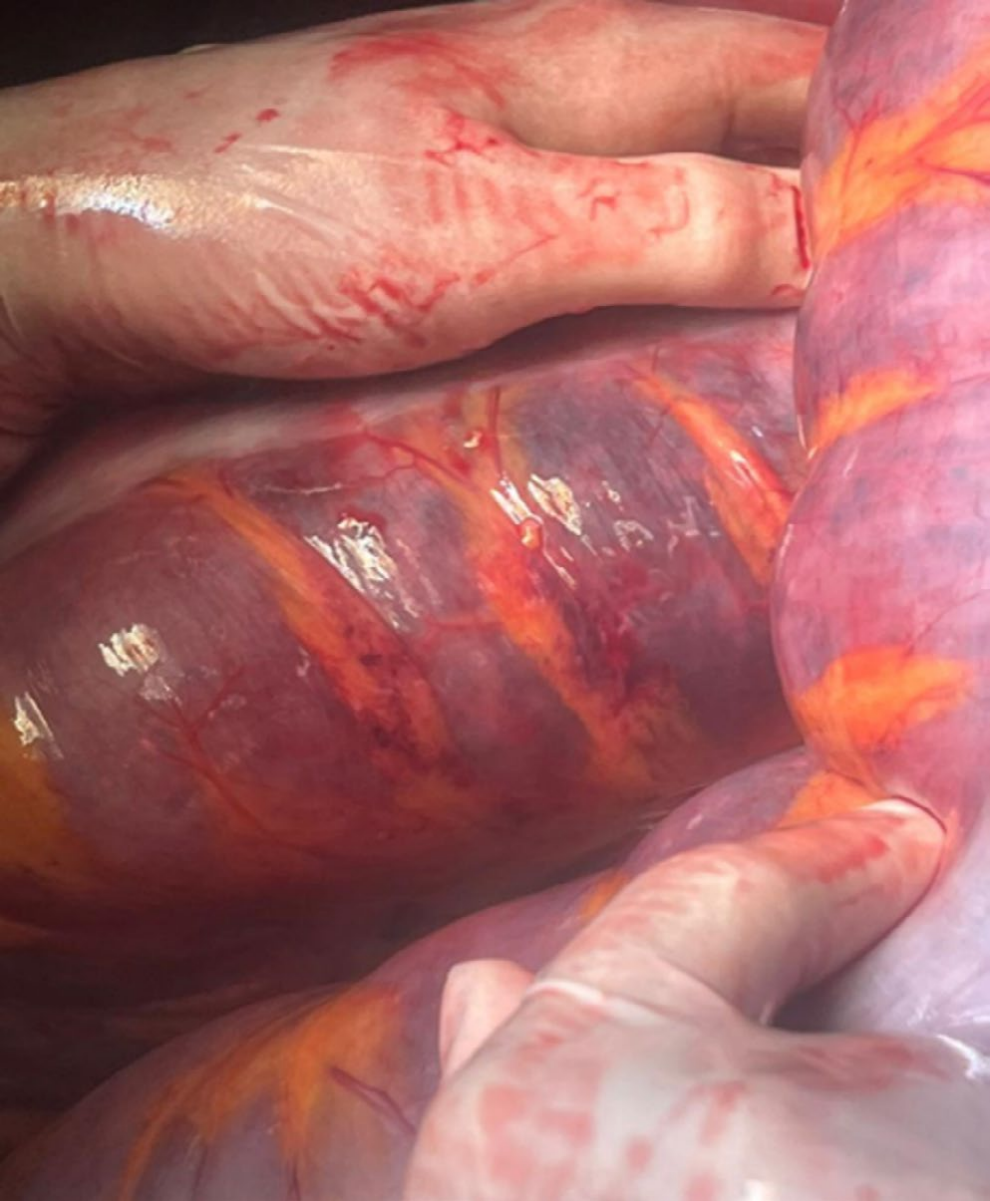
Shows patchy areas of purple‐appearing colon, which is consistent with tissue ischemia due to the process of the loop being rotated around its own axis. The infarcted section was resected with Hartman procedure.

Considering the clinical findings, the patient was highly suspected to have intestinal obstruction. Finally, the mother's condition persuaded clinicians to perform the CT scan with oral contrast. The CT scan showed a complete obstruction presumably in the distal part of small bowel and the patient was transferred to the operating room emergently; Figures 1, 2 and 3 show the radiological findings from plain abdominal x‐ray and two different views of contrast‐enhanced CT scan. A midline laparotomy incision was made; Intra‐operatively, the sigmoid colon was significantly dilated and purple‐appearing, as shown in Figures 4 and 5, which displays distended loops of sigmoid colon in the operation room. The gangrenous colon was resected and a Hartmann's procedure plus colostomy fixation was performed.

## 4 Conclusion and Results

The patient was transferred to the ICU for close observation. Her vital signs were within normal range. She received serum therapy, antibiotics, heparin and one pack cell transfusion due to a drop in hemoglobin level from 11.8 to 9.4. The fetus was assessed to be normal by frequent ultrasonographies and fetal heart rate monitoring.

Postoperatively, the patient experienced dyspnea and metabolic acidosis. She underwent echocardiography and cardiac workup by which the pulmonary thromboembolysis was ruled out. After respiratory physiotherapy and incentive spirometry, the dyspnea resolved. The patient and her fetus were otherwise well; she was discharged after 6 days with left colostomy and drainage tubes in her abdomen. Her further weekly follow/up showed normal condition of both mother and her fetus.

## 5 Discussion

As rare as it is, bowel obstruction can complicate a normal pregnancy. The most common etiologies for bowel obstruction are adhesions from previous abdominal surgeries that accounts for 60%–70% of cases, followed by volvulus (25%) and intussusception (5%). Adhesive bonds develop more commonly after open surgeries than laparoscopic surgeries as in our case who underwent two cesarean sections [9].

Volvulus, the second most common etiology, is a closed‐loop bowel obstruction, which develops when a bowel loop twists around the axis of its own mesentery more than 180° [10]. This phenomenon usually occurs in the second and third trimesters of pregnancy if ever. Several factors presumably play role in this anatomical abnormality; First, an enlarging uterus changes the anatomical location of bowel and colon. Second, the elevated level of progesterone and the release of relaxin during pregnancy makes the tissues more motile, which may lead to volvulus in a susceptible woman with congenital mal‐rotation or adhesions [11, 12]. Age and multigravidity are not reported as significant factors [4].

The prognosis is highly dependent on timely diagnosis and intervention. Clinical suspicion is warranted to avoid deferring diagnostic procedures and mistaking the diagnosis with other obstetric complications. According to a systematic review published in 2014, common presenting symptoms of bowel obstruction in pregnancy include abdominal pain (88%) and vomiting (67%), and clinical findings such as tenderness (49%) and distension (28%). However, laboratory findings are usually normal [13, 14]. The classic triad of generalized abdominal pain, vomiting, and obstipation should raise the clinical suspicion for midgut volvulus. The abdominal pain of volvulus usually transitions from colicky to constant in late stages of pregnancy and is mostly felt in the epigastrium [11]. Moreover, new onset back pain can occur due to an abdominal pathology during gestation [15].

As a matter of fact, the average time between the onset of symptoms until presentation of abdominal obstruction is about 48 h, since abdominal pain, nausea, and leukocytosis can occur in a normal pregnancy and cloud the clinical picture [6, 16]. According to literature review, almost all maternal deaths occur when patients seek medical intervention after 48 h following onset of symptoms. Maternal mortality in cases with viable bowel was 5% comparing to more than 50% in cases with bowel perforation, highlighting the importance of timely management [17]. One recent review study that assessed literature on midgut volvulus during pregnancy has reported that overall mortality rate of mother and fetus was 13% and 35%, respectively, and that maternal mortality all occurred in the third trimester [18].

The hesitation to perform a radiological procedure on mother is the first barrier in quick diagnosis. As available as it is, abdominal x‐ray fails to show the exact location of obstruction. Abdominal CT scan with contrast is another available method that is usually kept for essential conditions during pregnancy. This type of radiography, however, exposes the fetus to < 50 mGy that does not increase the risk of developing malformations, developmental delay, tumors, or genetic mutations to a significant amount. Nevertheless, transient hypothyroidism in a neonate who was exposed to contrast at 35‐week gestation has been reported. Hence, guidelines recommend monitoring of thyroid function in the newborn in this setting [3, 19].

Guidelines has warned about the cumulative radiation dose to the fetus during pregnancy to be < 5–10 rads. Generally, there is no single diagnostic procedure that exceeds five rads in total. The radiation dose of an abdominal radiograph is around 0.1–0.3 rads, while an abdominal and pelvic CT scan reaches up to five rads of fetal exposure. The most sensitive time for radiation exposure is the first week of gestation, which associates with the highest rates of fetal mortality, followed by the period between 10th and 17th weeks of gestation, when radiation may cause central nervous system teratogenesis. After that, the risk of childhood hematologic malignancy becomes the most important concern [20, 21]. Having said that, mother's health should always be the priority, since early diagnosis and intervention, yields better outcome than avoiding the radiation exposure in this context [16].

The type of intervention and the decision to cease the pregnancy is dependent on the gestational age, clinical scenario and the viability of the fetus. In the 1st trimester, the possibility of miscarriage should be explained to the mother before performing an endoscopic reduction or surgical resection. In the 2nd trimester, determining the best method of intervention is sometimes difficult, considering the fact that the mother's life is the priority, and the patient must be informed of probable delivery in the operation room. In the 3rd trimester, however, a cesarean section before bowel repair is usually the optimum intervention especially when the fetus is mature. There is no reason to end the pregnancy if the bowel is repaired, and if the mother is clinically capable of continuing the gestation [2, 5, 16].

Sigmoidectomy is the choice intervention in the acute setting when the colon appears to be nonviable or perforated [17]. When the bowel is evidently infarcted, the necrotic section must be resected and an anastomosis should be performed either in a one‐stage or a two‐stage procedure, depending on the patient's physiology. Importantly, extensive surgical resection might have complications like short gut syndrome, which mandates life‐long total parenteral nutrition for the patient [22]. Detorsion of the volvulus by colonoscopy and decompression of bowel by placement of a soft rectal tube may be sufficient when there is no evidence of infarction or acute abdomen mainly in the first trimester. Noteworthy to say, performing colonoscopic detorsion in late pregnancy is rarely successful [23].

Management of bowel obstruction in pregnancy necessitates a multidisciplinary intervention and prompt clinical decision‐making. The hesitation to perform radiological procedures defers timely management and should be avoided. Patient should undergo the safest available diagnostic procedure (either CT or MRI) and shall be managed accordingly as soon as possible. The lack of MRI in our center was one limitation of this study, however as mother's life was the priority; the patient underwent a contrast‐enhanced CT scan and emergently was managed surgically. The outcome of the patient and her fetus in our study was promising; however, longer follow‐up is necessary to draw a definite conclusion about the management of this patient.

## Author Contributions

**Naghmeh Kian:** writing – original draft, writing – review and editing. **Atefeh Moridi:** resources, writing – review and editing.

## Consent

All participants in this study were informed about the process of writing and consented to the publication. Written Informed consent has been obtained.

## Conflicts of Interest

The authors declare no conflicts of interest.

## Data Availability

Data sharing is not applicable to this article as no datasets were generated or analyzed during the current study.
